# De Novo Genome Assembly of *Toniniopsis dissimilis* (Ramalinaceae, Lecanoromycetes) from Long Reads Shows a Comparatively High Composition of Biosynthetic Genes Putatively Involved in Melanin Synthesis

**DOI:** 10.3390/genes15081029

**Published:** 2024-08-05

**Authors:** Julia V. Gerasimova, Andreas Beck, Agnes Scheunert, Om Kulkarni

**Affiliations:** 1 Department of Lichenology and Bryology, Botanische Staatssammlung München, SNSB-BSM, 80638 Munich, Germany; 2 Senckenberg Research Institute and Natural History Museum, 60325 Frankfurt am Main, Germany; julia.gerasimova@senckenberg.de; 3 Systematics, Biodiversity and Evolution of Plants, Faculty of Biology, Ludwig-Maximilians-Universität München, 80638 Munich, Germany; 4 GeoBio-Center, Ludwig-Maximilians-Universität München, 80333 Munich, Germany; 5 Genomics Core Facility, Staatliche Naturwissenschaftliche Sammlungen Bayerns, SNSB-GCF, 80638 Munich, Germany; scheunert@snsb.de (A.S.); kulkarni@snsb.de (O.K.)

**Keywords:** lichen, symbiosis, genomics, type I polyketide gene, ecology, long-read sequencing, fungal RiPP-like proteins

## Abstract

Lichens have developed numerous adaptations to optimize their survival in various environmental conditions, largely by producing secondary compounds by the fungal partner. They often have antibiotic properties and are involved in protection against intensive UV radiation, pathogens, and herbivores. To contribute to the knowledge of the arsenal of secondary compounds in a crustose lichen species, we sequenced and assembled the genome of *Toniniopsis dissimilis*, an indicator of old-growth forests, using Oxford Nanopore Technologies (ONT, Oxford, UK) long reads. Our analyses focused on biosynthetic gene clusters (BGCs) and specifically on Type I Polyketide (T1PKS) genes involved in the biosynthesis of polyketides. We used the comparative genomic approach to compare the genome of *T. dissimilis* with six other members of the family Ramalinaceae and twenty additional lichen genomes from the database. With only six T1PKS genes, a comparatively low number of biosynthetic genes are present in the *T. dissimilis* genome; from those, two-thirds are putatively involved in melanin biosynthesis. The comparative analyses showed at least three potential pathways of melanin biosynthesis in *T. dissimilis*, namely via the formation of 1,3,6,8-tetrahydroxynaphthalene, naphthopyrone, or YWA1 putative precursors, which highlights its importance in *T. dissimilis*. In addition, we report the occurrence of genes encoding ribosomally synthesized and posttranslationally modified peptides (RiPPs) in lichens, with their highest number in *T. dissimilis* compared to other Ramalinaceae genomes. So far, no function has been assigned to RiPP-like proteins in lichens, which leaves potential for future research on this topic.

## 1. Introduction

Lichens represent a symbiosis composed of at least one main fungal (mycobiont) and one main algal (photobiont) partner with a diverse group of life forms named after the main fungal partner [1]. Since this dual nature of lichens was discovered [2], it has been recognized that, in fact, they even represent ecosystems accommodating many additional organisms, such as bacteria, viruses, and other fungi (e.g., [3,4,5]). This symbiotic nature is not visible at first glance and results in diverse morphological appearances, commonly divided into three major growth forms: crustose, foliose, and fruticose [6]. The mycobionts—defined by their nutrition strategy—form a polyphyletic group, overarching many fungal classes and even divisions [1]. Recent advances in the genomic and transcriptomic analyses of lichen-forming fungi (e.g., [7,8,9,10,11,12,13,14,15]) significantly expanded the knowledge of lichens, tempting the announcement of the ‘coming golden age for lichen biology’ [16]. Nevertheless, the number of assembled lichen fungal genomes is still rather low compared to over 5000 fungal genomes available at the National Center for Biotechnology Information (NCBI): https://www.ncbi.nlm.nih.gov/ (accessed 10 June 2024) and the Joint Genome Institute (JGI): https://genome.jgi.doe.gov/portal/ (accessed 10 June 2024) web portals. Amongst these, the major part is from more prominent species of foliose and fruticose lichens, compared to inconspicuous crustose species in the broad sense, although they account for about 70% of lichen-forming fungi [1].

The recently described crustose species *Toniniopsis dissimilis* Gerasimova and A. Beck [17] belong to the large family Ramalinaceae, which contains 40 genera and exhibits various growth types ranging from crustose to fruticose. Members of this family also occupy a wide range of ecological niches [18], exhibiting a high diversity of lichen secondary compounds ([11] and references therein). Currently, there are six published genomes from the Ramalinaceae family with four fruticose *(Niebla homalea* (Ach.) Rundel and Bowler, *Ramalina farinacea* (L.) Ach., *Ramalina intermedia* (Delise ex Nyl.) Nyl., and *Ramalina peruviana* Ach.; [19,20]), and two crustose species (*Bacidia gigantensis* Lendemer, McCune, and McMullin, and *Bacidia rubella* (Hoffm.) A. Massal.; [17,21]). This fact allowed us to put the diversity of biosynthetic gene clusters (BGCs) in the context of growth types and ecology of the family. Recent studies have revealed an unexpected abundance of Type I Polyketide genes (T1PKS) in the crustose lichen *B. rubella* [11]. This evidence raised the question of whether we could observe a similar pattern in another crustose member of the same family.

*Toniniopsis dissimilis* has been found in various countries across Eurasia, typically in high elevations (areas above 1000 m asl.) or in high latitudes, suggesting that it may be well-adapted to colder climates but also higher ultraviolet (UV) radiation [17]. This study aims to contribute to genomic research on lichen-forming fungi, using a reference genome of *T. dissimilis* based on long reads in the comparative genomic framework of other published genomes from the Ramalinaceae family. Within the T1PKS, we specifically focused on the BGCs putatively involved in the production of melanins in *T. dissimilis*, as those represent four out of the six identified T1PKS genes (see Results). Melanins are of interest due to their potential role in stress tolerance and their presence in lichens from diverse habitats, especially those with high levels of abiotic stress, such as polar and montane regions.

Dark melanin pigments have been observed across all domains of life, and all fungal phyla comprise species that can produce melanin. The presence and potential role of melanin pigments in lichenized ascomycetes have been extensively discussed by Mafole et al. [22]. The authors state that melanins are almost certainly involved in the tolerance of lichens to a variety of abiotic stresses in addition to light and heat, strongly suggesting that they play a crucial role in stress tolerance by protecting the mycobiont from high UV radiation and the photobiont from high photosynthetically active radiation (PAR). Chemically, melanins are challenging to define due to their complex structure, but in fungi, they typically belong to either the eumelanin or the allomelanin group ([22] and references therein). Despite playing an important role, the biosynthetic pathways of lichen melanin biosynthesis and their full spectrum of functions still need to be explored and confirmed by further work, which we intend to contribute by analyzing the occurrence of BGCs putatively involved in melanin biosynthesis of lichen-forming fungi.

## 2. Materials and Methods

### 2.1. Sample Collection, DNA Extraction, and Sequencing

**Figure 1:**
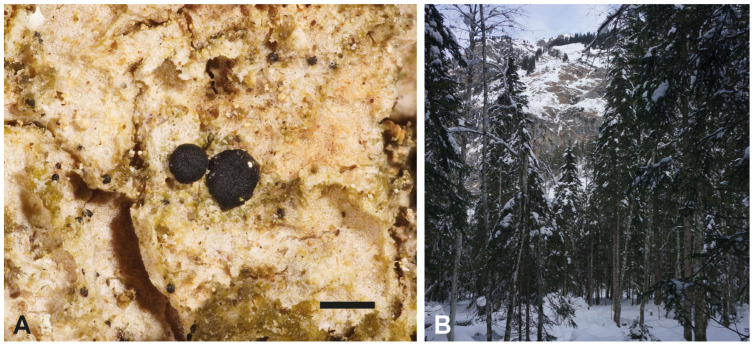
(**A**) Detail of *T. dissimilis* (M-0355157): thallus, consisting of scattered, rounded to slightly flattened or subsquamulose granules, and dark apothecia. Scale: 0.5 mm. (**B**) Habitat of *T. dissimilis*: A mixed forest in the Oytal Valley, Allgäu (Bavaria, Germany), 47°23′08″ N 10°20′35″ E, ca. 1080 m asl.

*Toniniopsis dissimilis* was collected in February 2023 in the Oytal Valley (Bavaria, Germany) from the bark of the trunk of the same *Fraxinus* tree, from which the type material had also been collected (Figure 1). The specimen is deposited in the Botanische Staatssammlung München, SNSB-BSM, with the herbarium number M-0355157. Macrographs of external characters were taken on a Leica Z6 Apo microscope (with a 2.0× Planapo lens; Leica, Wetzlar, Germany) with a Sony α 6400 camera (Sony, Tokyo, Japan) attached and equipped with a StackShot Macro Rail (Cognisys, Traverse City, MI, USA). A single image was prepared from 60 serial images using Helicon Focus v.7 software (Helicon, Kharkov, Ukraine).

About 100 apothecia were selected under a compound microscope (Leica Z6 Apo; Leica, Germany) with sterile tweezers, and the DNA was extracted as described in Gerasimova et al. [17] using the Stratec Invisorb Spin Plant Mini Kit (Stratec Molecular GmbH, Berlin, Germany) following the manufacturer’s instructions.

Nanopore library prep was performed using 700 ng of input DNA and the ONT Ligation Sequencing Kit V14 (SQK-LSK114) following the manufacturer’s protocol (version “revJ” from 29 June 2022) except generally prolonged incubation times. A total of 18 fmol of the library was sequenced on two R10.4.1 MinION flow cells on a MinION Mk1B sequencer (ONT).

### 2.2. Genome Assembly and Filtering

Raw data were re-basecalled with guppy v6.5.7 (ONT) using the fast (FAST), high-accuracy (HAC), and super accuracy (SUP) basecalling models; the resulting reads were compared, and the best data set was chosen for further analysis. Quality control was conducted using MinIONQC v1.4.2 [23], the *readlength.sh* script from the BBTools package v39.01 (Bushnell B, https://sourceforge.net/projects/bbmap), toulligQC v2.4 (GenomiqueENS core facility, https://github.com/GenomiqueENS/toulligQC), fastQC v0.12.1 (Babraham Bioinformatics, https://www.bioinformatics.babraham.ac.uk/projects/fastqc/), multiQC v1.15 [24], and nanoQC v0.9.4 and NanoStat v1.6.0, both from the NanoPack software package [25]. The leftover adapter sequence and minimum length filtering was removed by chopper v0.6.0 (NanoPack).

The reads were assembled using Flye v2.9 with the *--nano-hq* and *--meta* options [26]. The resulting assembly underwent polishing with Medaka v1.11.3 (https://github.com/nanoporetech/medaka), utilizing the *medaka_consensus* command with the basecalling model specified as *-mr1041_e82_400bps_hac_g632*.

The metagenome assembly was employed to retrieve contigs belonging to our phylum of interest. Blobtools v1.1 [27] was used for taxonomic classification. Initially, Minimap2 calculated read coverage, which was then converted to the Blobtools format using *blobtools map2cov*. We followed the double-blast approach detailed by [14] (https://github.com/theo-llewellyn/TeloschistalesMetagenomics). Taxonomic classification was performed using the results from BLAST v2.15.0+ [28] and DIAMOND v2.1.9.163 [29].

Results from Blobtools were utilized to quantify and visualize taxonomic identification, read coverage, and GC content. Contigs assigned to Ascomycota, with a minimum coverage of 60×, were extracted from the Blobtools table as described in [21].

The Flye assembly was further polished with the filtered short (150 bp) Illumina reads obtained from the same type locality using Polypolish v0.6.0 [30] and POLCA v4.0.5 [31], following the Trycycler workflow (https://github.com/rrwick/Trycycler/wiki/Polishing-after-Trycycler). The polished long-read assembly was compared with all existing Ramalinaceae genomes using QUAST v5.2.0 [32] and BUSCO fungi_odb9 [33] (See Table 1 in Section 3).

### 2.3. Annotation

The assembly was annotated using Funannotate v1.8.16 (https://github.com/nextgenusfs/funannotate). Standard steps for cleaning repetitive contigs and masking repeats were followed. For the funannotate predict step, all default ab initio and evidence-based predictors were used. To improve the protein prediction step, we provided additional transcript evidence from the assembled *Toniniopsis* transcripts (data are currently being prepared for publication) and protein evidence from *B. rubella* (GCA_032158265.1) and *B. gigantensis* (GCA_019456465.1). Biosynthetic gene clusters were annotated with antiSMASH v7.1.0 [34], and the final GenBank files were generated. Due to the incompatibility of the Funannotate v1.8.16 file with the newest version of the antiSMASH output format, the resulting .gbk files were converted to .gff using the genbank_to script (https://pypi.org/project/genbank-to/) and were used for further downstream analysis on 27 lichen genomes.

### 2.4. Phylogenetic Analysis

For subsequent phylogenetic analysis, we prepared an alignment based on the ketoacyl synthase (KS) annotated domain sequences from the T1PKSs. We included the predicted KS amino acid alignment reported by Kroken et al. [35] to compare the tree topology published therein and the large phylogeny of the T1PKS in the previous study [11]. As the large phylogenetic tree revealed that four out of six KS genes from *T. dissimilis* were nested in the putative melanin clade, we selected the characterized KS domains from the melanin BGC from *Pyricularia oryzae* (BGC0002154) and *Glarea lozoyensis* (BGC0001258) as a query against the 687 KS genes obtained from the presented analysis of 27 lichen genomes to reconstruct the phylogeny based on the putative melanin homologous genes from the lichen-forming fungi. To search for homologous genes, we used the SWIPE pipeline with the following parameters: maximum number of hits of 20 with a minimum bit score of 20 (https://bitbucket.org/yangya/phylogenomic_dataset_construction/src/master/bait_homologs.py), which resulted in a dataset comprised of 497 sequences. The dataset was aligned using MAFFT v7.525 [36] with the following parameters: *--globalpair --maxiterate 1000*, which correspond to the G_INS_i strategy, suitable for the conservative regions such as the KS domain. The alignment was trimmed using trimAl v1.4.rev15 [37] with the *gappyout* mode, which removes poorly aligned regions. The quality of the final alignment was verified manually. We selected the best-fitting substitution model (LG+R9) according to the Bayesian Information Criterion using ModelFinder [38]. We calculated a phylogenetic tree using maximum-likelihood (ML) analysis implemented in IQ-TREE v2.1.4 [39], with 1000 ultrafast bootstrap replicates [40]. After the first phylogeny reconstruction, three sequences failed the composition chi2 test (*p*-value < 5%; df = 19) as they contained more than 50% gaps/ambiguity; therefore, we removed them from the final alignment. The resulting tree was visualized using FigTree v1.4.3 and a custom R script, with additional annotations added in Adobe Illustrator v28.5.

### 2.5. Identification of the Fungal RiPP-like Orthologues Using OrthoFinder

Ribosomally synthesized and posttranslationally modified peptides (RiPP)-like proteins extracted from the .gff files were searched against annotated proteins from the UniProtKB/Swiss-Prot database using BLASTp. The protein sequences with at least 20% similarity were retrieved for further analysis. To identify orthologous genes, we inferred orthogroups with Orthofinder v2.5.5 [41] for all extracted RiPPs, including the best hits from the database. The Markov Cluster (MCL) inflation parameter was left at the default (1.5). The trees utilized by OrthoFinder were reconstructed using Fasttree v2.1.10 (*m msa, -A mafft*, and *-S diamond*) [42].

## 3. Results and Discussion

### 3.1. Library Prep and Sequencing

The Nanopore library had a final library peak at approximately 14 Kb with a very broad fragment length distribution. The re-basecalled dataset comprised 9.9–10 million reads and 9.8–9.9 gigabases, depending on the basecalling model. For further analysis, the HAC dataset with a minimum quality of Q9 was chosen because of higher amounts of passed reads at a generally higher quality than with the FAST model, alongside a higher proportion of passed very long reads than the SUP model. The processed, filtered dataset used for assembly had 6.5 Gb of data and 6.6 million reads with a read length N50 of 1581 bp and a mean read quality of Q 12.9.

### 3.2. General Characteristics of the De Novo Toniniopsis dissimilis Genome

General metrics of the newly obtained genome data of *T. dissimilis* compared with other members of the family Ramalinaceae are given in Table 1.

**Table 1:** Comparison of seven genome assembly metrics from the Ramalinaceae family.

	*Toniniopsis dissimilis*	*Bacidia* *gigantensis*	*Bacidia* *rubella*	*Niebla* *homalea*	*Ramalina* *farinacea*	*Ramalina* *intermedia*	*Ramalina* *peruviana*
**Assembly Size (Mb)**	29.22	33.11	33.7	50.56	32.74	26.24	27
**Largest Scaffold (bp)**	1,545,693	3,530,911	2,353,056	3,158,485	2,450,536	898,913	694,821
**# Scaffolds**	85	24	246	52	44	196	1657
**N50 (bp)**	741,547	1,807,239	1,771,855	1,266,640	1,546,935	273,318	40,431
**GC content**	47.33%	44.67%	45.25%	37.96%	46.69%	51.89%	50.66%
**# Genes**	10,058	9207	9424	8586	8640	8033	7133
**# Proteins**	10,011	9158	9365	8521	8575	7982	7080
**# tRNA**	47	49	59	65	65	51	53
**Unique Proteins**	2463	1919	1781	985	789	387	598
**Prots at least one ortholog**	7422	7135	7512	7501	7774	7592	6467
**Single-copy orthologs**	2530	2462	2462	2462	2462	2462	2462
**Number of BGCs**	47	39	54	77	72	71	51

**Figure 2:**
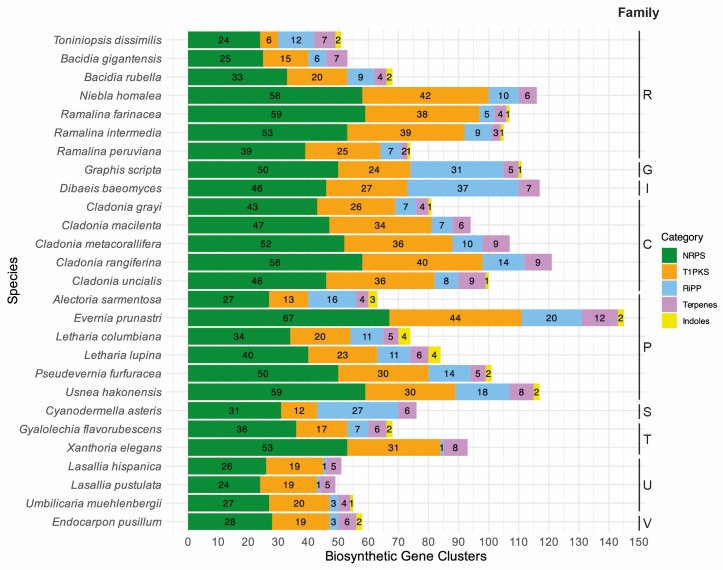
Diverse classes (categories) of secondary metabolites annotated for the selected lichen-forming fungi are given in color with the color code on the right-hand side. The total number of biosynthetic gene clusters is given on the *x*-axis, and the species names are on the *y*-axis. The following single letter code indicates families: C: Cladoniaceae, G: Graphidaceae, I: Icmadophilaceae, P: Parmeliaceae, R: Ramalinaceae, S: Stictidaceae, T: Teloschistaceae, U: Umbilicariaceae, and V: Verrucariaceae.

The final de novo assembly of *T. dissimilis* had a size of 29.22 Mb assembled in 85 scaffolds with an N50 of 741.5 Kb and 98.6% BUSCO completeness (fungi_odb9). Using multiple ab initio gene-calling methods, as mentioned in the Materials and Methods, 10,058 genes were identified, which is higher than all other Ramalinaceae genomes (Table 1). The *Toniniopsis* genome contains 44 BGCs, similar to *B. gigantensis* with 39 BGCs but relatively lower than other Ramalinaceae genomes. *Bacidia rubella* contains 54 BGCs, *R. farinacea*, *R. intermedia*, and *R. peruviana* contain 72, 71, and 51 BGCs, respectively, and *N. homalea* contains the highest number at 77 BGCs (Table 1, Figure 2).

### 3.3. Phylogenetic Analyses of the KS Domain of T1PKS Genes of T. dissimilis

To place the putative biosynthetic compounds of T1PKS genes of *T. dissimilis* in a larger phylogenetic context, we reconstructed a phylogeny based on the KS domains of 27 lichen genomes as in Gerasimova et al. [11], including 80 KS domains from Kroken et al. [35]. *Toniniopsis dissimilis* contained 47 BGCs, of which six are T1PKS (Table 2, Figure 2). The total T1PKS biosynthetic arsenal is much lower compared to other Ramalinaceae genomes: *B. gigantensis* and *B. rubella* contained 15 and 20 T1PKSs, *R. farinacea*, *R. intermedia*, and *R. peruviana* contain 34 T1PKSs in average, and *N. homalea* contained the highest number at 42 T1PKSs, respectively (Figure 2).

According to the antiSMASH annotation, four out of six T1PKS BGCs are potentially involved in the biosynthesis of melanin in *T. dissimilis,* further strengthening the assumption of Mafole et al. [22] and Gerasimova et al. [11] that melanins have an essential role in lichens. Specifically, the *tondispred_002824* gene has a similarity score of 71% to 1,3,6,8-tetrahydroxynaphthalene T1PKS biosynthetic genes of *Glarea lozoyensis* (BGC0001258), *Pyricularia oryzae* (BGC0002154), and *Nodulisporium* sp. (BGC0001257), while the *tondispred_004340* gene has 69% similarity to 1,3,6,8-tetrahydroxynaphthalene biosynthetic gene from *G. lozoyensis* (BGC0001258) and other fungi.

The *tondispred_001459* gene has 100% similarity to naphthopyrone T1PKS biosynthetic gene of *Aspergillus nidulans* (BGC0000107), and the *tondispred_004406* gene has 100% similarity to YWA1 from *Aspergillus oryzae* (BGC0002175) and naphthopyrone from *Aspergillus nidulans* (BGC0000107).

**Figure 3:**
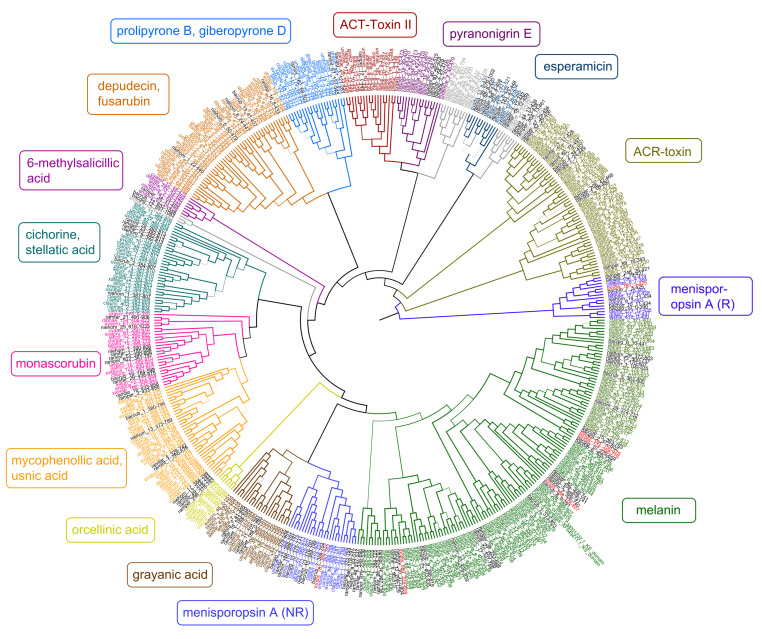
Maximum-likelihood phylogeny of ketoacyl synthase (KS) genes from Type I PKS genes as inferred by IQ-TREE. Genes from *T. dissimilis* are marked in red, and other Ramalinaceae genomes are in black. The putative secondary compounds clades are given in color boxes, with the largest clade assigned to melanins (green). Clades where no clear annotations could be found are in grey.

As there were only a few predicted melanin T1PKS from *Bacidia* species in the previous study, only two KS genes of *T. dissimilis* could be analyzed in the context of reducing (R) and non-reducing (NR) T1PKS groups as defined by Gerasimova et al. [11], which did not belong to putative melanins. The *tondispred_004967* (*tondis_787*:3-425) was nested together with *bacrubpred_003664* (*bacrub_7*:3-425) corresponding to R-I *PKS7* group with lovastatin/citrinin/diketide compounds known to be produced in this group (Figure 3). The second gene from the same BGC—*tondispred_004968* (*tondis_787*:368-832) was nested together with *bacrubpred_003665* (*bacrub_7*:356-704), which corresponded to NR-I *PKS13* group (orsellinic acid, zearalenone) [11] (Figure 3).

### 3.4. Putative Melanin Clades That Include B. gigantensis, B. rubella, and T. dissimilis

Since four of the six KS genes of *T. dissimilis* were assigned to the putative melanin clade, we start the discussion with these genes. To reconstruct the phylogeny of the putative melanin KS from the lichen-forming fungi, we selected the characterized KS domains from the melanin BGC from *P. oryzae* (BGC0002154) and *G. lozoyensis* (BGC0001258) as a query against the 687 KS genes obtained from the presented analysis of 27 lichen genomes to search for homologous genes. The results indicate that several gene copies of KS are putatively involved in melanin biosynthetic production and are present in *Toniniopsis* and both *Bacidia* species (Figure 3, clade highlighted in green).

### 3.5. Clade with *bacrubpred_003614 (bacrub_6)*, *tondispred_004406 (tondis_774)*, and *bacgigpred_005712 (bacgig_10)*

These putative melanin biosynthetic genes from *T. dissimilis* and *Bacidia* species formed one clade (partly corresponding to NR-II in Gerasimova et al. [11]). All three species reveal the same PKS domain structure: SAT-KS-AT-PT-ACP-PP, with 93% similarity for the KS domain and 77% to 90% for all other domains. In addition, all three BGCs contained a gene coding for the Major Facilitator Superfamily (MFS1) protein and Aflatoxin regulatory protein (AflR) with 63–75% and 38–46% similarity, respectively. The gene composition of the clusters differed otherwise.

Both genes from *Toniniopsis* and *Bacidia* showed 70% similarity to *P. oryzae* melanin BGC (BGC0002154). Zhu et al. [43] argued that melanin is necessary for *P. oryzae* for appressorium turgor, penetration, and virulence, while in lichen-forming fungi, melanin likely plays an essential role as a sunscreen and herbivore deterrent (See details below).

### 3.6. Clade with *bacrubpred_007628 (bacrub_15)*, *tondispred_001459 (tondis_191)*, and *bacgigpred_001679 (bacgig_2)*

These putative melanin biosynthetic genes from *Toniniopsis* and *Bacidia* species similarly formed one clade, sister to the abovementioned clade. This clade showed 71–72% similarity to the YWA1 biosynthetic cluster from *Aspergillus oryzae* (BGC0002175) and naphthopyrone from *A. nidulans* (BGC000107), with the next closest hit to *Pyricularia oryzae* melanin BGC (BGC0002154) and *G. lozoyensis* (BGC0001258) with 69% similarity.

All three species revealed the same PKS domain’s structure but, compared to the previous clade, had an additional thioesterase domain, i.e., SAT-KS-AT-PT-ACP-PP-TE. The similarity within the KS domains is between 89% to 94%, and all other domains are 79% to 96% similar. The gene composition of the clusters otherwise differed.

### 3.7. Other Putative Melanin Genes from Two Bacidia Species

There were three other putative melanin genes from *Bacidia* species present in the large melanin clade: bacrub_5 (*bacrubpred_002956*, corresponds to NR-IV aflatoxin clade in [11]), bacrub_1 (*bacrubpred_000208*, corresponds to NR-II melanin clade in [11]), and bacgig_8 (*bacgigpred_005093*, annotated here for the first time). All three genes had the highest similarity to the KS genes encoding YWA1, naphthopyrone, or 1,3,5,8-tetraxydroxynaphthalene.

Interestingly, in our previous study [11]—using the older software versions—only one melanin biosynthetic gene was annotated for two *Bacidia* species.

The KS of *B. rubella* from this cluster showed 70% BLAST identity with *bacrubpred_000202* from the previous study, where it was nested together with *bacgigpred_008278* in the NR-II group (melanins) and sister to the clade which contained *G. lozoyensis PKS1*. However, *bacrubpred_000202* showed 98% similarity with *bacrubpred_000208* (*bacrub_1*:373-803), which also has 70% similarity with *P. oryzae* melanin T1PKS (BGC0002154), but it did not form a clade with other members of Ramalinaceae. Similarly, *tondis_002824* (*tondis_288*) had a 70–71% similarity to *P. oryzae* melanin T1PKS (BGC0002154).

### 3.8. YWA1: Alternative Precursor for the Melanin Biosynthesis

In several cases, the second-best hit (next to 1,3,6,8-tetrahydroxynaphthalene) was YWA1 from *Aspergillus oryzae* (BGC0002175). YWA1 is a known melanin precursor in *A. nidulans* and *A. fumigatus*, being converted to DHN melanin [44,45]. *PksP* of *A. fumigatus* is involved in synthesizing the yellow molecule YWA1. However, the biosynthetic transformation to melanin is not necessarily possible as BLAST with DHN melanin pathway components from *A. fumigatus* (i.e., GenBank accessions E9QUT3, Q4WZB3 and Q4WZB4) revealed their absence from the *P. variotii* genome [45]. Thus, *PvpP* (the *PksP* homolog of *P. variotii*) is required to synthesize the characteristic yellow pigment observed for petri dish cultures, but without the additional modifications seen in other Eurotiales species [45].

The closest hit of several biosynthetic genes from *Toniniopsis*—especially *tondispred_004406* (*tondis_774*)—to the YWA1 reveals the potential for the various ways of melanin biosynthesis. Similarly, a high capacity for melanin biosynthesis was revealed in *Bacidia* (Figure 3). However, experimental proof is necessary to explore these pathways for melanin biosynthesis in lichen-forming fungi.

### 3.9. An Ecological Perspective of Multiple Copies of Putative Melanin Genes in the Crustose Ramalinaceae

Melanins are a diverse group of substances that play a role in virulence, morphogenesis, or the response to environmental stress and can be synthesized via different pathways [22,46]. It is well known that melanins are dark pigments produced by fungi and other organisms and are frequently concentrated in the cell wall in fungi [44]. The melanins are composed of several types of phenolic monomers and are often complexed with protein and, less often, carbohydrates [47,48]. The nomenclature of melanins in fungi is based on their composition and their biosynthesis pathway and includes 1,8-dihydroxynaphthalene (D2HN) melanin, catechol melanin, dihydroxyphenylalanine melanin, and γ-glutaminyl-4-hydroxybenzene melanin [46]. The best-characterized fungal melanin is likely D2HN melanin, which is synthesized by polyketide pathways. The starting reaction with head-to-tail joining and cyclization of one acetyl-coenzyme A (acetyl-CoA) molecule and four malonyl-CoA molecules, or only of malonyl-CoA molecules, is catalyzed by an iterative T1PKS and leading to the formation of 1,3,6,8-tetrahydroxynaphthalene (T4HN) [49]. This process is followed by several consecutive enzyme-catalyzed steps producing D2HN and building up melanin by polymerization due to oxidase/laccase reaction [48,50,51]. Next to D2HN melanin, PKSs produce deoxybostrycoidein melanins as well [52]. Consequently, 1,3,6,8-tetrahydroxynaphthalene and naphthopyrone are major putative precursors for melanin biosynthesis [44,53].

All the putative melanin genes from the 27 lichen genomes showed a major similarity to the biosynthetic genes encoding for the 1,3,6,8-tetrahydroxynaphthalene, naphthopyrone, and YWA1 biosynthesis. The occurrence of genes whose closest homologs are involved in synthesizing melanin and melanin precursors in the large melanin clades (Figure 3, green clade) is striking. It indicates an important role in melanin production, particularly in the crustose species of the Ramalinaceae.

*Toniniopsis dissimilis* and both *Bacidia* species are epiphytic and occur in semi-exposed habitats. *Bacidia rubella,* however, is often found in the sunlight forest edges or even more sun-exposed habitats. *Bacidia gigantensis* and *B. rubella* have 15 and 20 T1PKS genes annotated versus six T1PKS genes in *T. dissimilis*. While in *Bacidia* species, these T1PKSs may have various functions as they were nested in different groups in the phylogeny (Figure 3), *Toniniopsis* revealed a high number of putative melanin genes.

Most putative melanin genes showed high similarity to *P. oryzae* melanin T1PKS (BGC0002154). Zhu et al. [43] argued that melanin is necessary for *P. oryzae* for appressorium turgor, penetration, and virulence, while in lichen-forming fungi, melanin might play an essential role in defense mechanisms contributing to the survival of lichens under exposure to UV radiation [54]. Given the existence of albino mutants, melanins may not always be essential for growth and development but enhance the survival and competitive abilities of fungi in certain environments [22,46]. This may also be true for *T. dissimilis*, which sometimes forms pale-colored to almost colorless apothecia (own observation).

### 3.10. Putative ‘Menisporopsin A’ Biosynthetic Gene Cluster

Our phylogenetic results showed that two biosynthetic genes from the same BGC from *B. rubella* and *T. dissimilis* were nested in two clades in the phylogeny: R-PKS (*bacrub_7*:3-425 and *tondis_787*:3-425) and NR-PKS (*bacrub_7*:356-704 and *tondis_787*:368-832). The NR-PKS genes formed a separate clade outside of the melanin clade, with the closest hit to the oronofacic acid BGC from *Pseudomonas syringae* (BGC0000041) and pyranonigrin E from *Aspergillus niger* (BGC0001124). The BLASTp search against the UniProtKB/Swiss-Prot database showed a high similarity of both R- and NR-PKS KS domains against A0A6F9DXA0.1 (R-PKS, *men1*) and A0A6F9DYX9.1 (NR-PKS, *men2*) isolated from *Menisporopsis theobromae*, involved in the biosynthesis of Menisporopsin A.

Menisporopsin A is a bioactive macrocyclic polylactone produced by the fungus *M. theobromae* BCC 4162 [55]. The same authors proposed a scheme for the biosynthesis of this compound, in which reducing and non-reducing polyketide synthases would catalyze the formation of each Menisporopsin A subunit while an additional non-ribosomal peptide synthetase (NRPS)-like enzyme would be required to perform multiple esterification and cyclolactonization reactions [55].

The similarity between *B. rubella* and *T. dissimilis* domains was high, from 84% to 95% for R-PKS and 70% to 90% for NR-PKS. The comparison of all other domains with *men1* and *men2* also showed high percent similarity in a range from 53 to 79% for NR-PKSs and 46 to 79% for R-PKSs. The similarity between the lichen KS domains was high, ranging from 69 to 95% for R-PKSs and 56 to 90% for NR-PKSs. A striking similarity with the presence of R-PKS and NR-PKS in the same BGC with high similarity to *M. theobromae* allows us to speculate that they may be involved in the biosynthesis of the same product.

### 3.11. Fungal RiPP-Like Proteins

The RiPP-like proteins were first described in 2007 with the discovery of amanitin [56]. Four classes of RiPPs are currently recognized in fungi: (1) amatoxins, (2) borosins, (3) dicaritins, and (4) epichlöecyclins [56]. The ecological role of RiPP-like proteins in nature is still unknown, as research has focused on pharmaceutical benefits and applications in medicine. So far, characterized groups of RiPP-like proteins are known from Agaricomycetes (amatoxins and borosins), Eurotiomycetes (dicaritins), and Sordariomycetes (dicaritins and epichlöecyclins). The highest number of RiPP-like proteins in the genomes analyzed here were annotated for *Dibaeis* and *Graphis* (above 30), while *Toniniopsis* had the highest number of RiPP-like proteins within Ramalinaceae (Figure 2). However, to the best of our knowledge, there are no characterized RiPP-like proteins from Lecanoromycetes [56]. To assign the RiPP-like genes to a putative function, we implemented BLASTp search against the characterized genes in the UniProtKB/Swiss-Prot database for the closest hits. The best hits with at least 20% similarity were used together with the lichen RiPP-like proteins to assign them to orthogroups using OrthoFinder. The orthogroups containing lichen RiPP-like proteins did not contain non-lichen RiPP-like proteins. Given this evidence, no putative function could be assigned to the 12 RiPP-like proteins of *Toniniopsis* nor the other lichen RiPP-like proteins, which leaves potential for future research on this topic.

## 4. Conclusions

The newly generated genome sequence of *T. dissimilis* with 98.6% BUSCO completeness provides a robust basis for analyzing biosynthetic gene clusters in this indicator species of old-growth forests. Compared to the other genomes of the family Ramalinaceae, it contained the lowest number of T1PKS genes. Fruticose members of this family (*Niebla* and *Ramalina* species) contain higher numbers of T1PKS genes as compared to the crustose ones (*Toniniopsis* and *Bacidia* species), namely from 25 to 42 as compared to 6 to 15 (Figure 2). Four of the six T1PKS genes in *Toniniopsis dissimilis* are potentially involved in the formation of melanin, suggesting an important function of this dark-colored pigment in this species. Melanin is known to act as a photoprotective agent for both the myco- and photobiont and reduce herbivory [22]. The fact that the fruticose members of Ramalinaceae showed a higher composition of BGCs and T1PKS genes, particularly, leads us to conclude that they may play an essential role and can be linked to the ecology of those species.

*Toniniopsis dissimilis* and *B. gigantensis* are epiphytic and grow in semi-exposed habitats. An epiphytic *B. rubella*, in contrast, can occur in more dry and sunny-exposed habitats. *Niebla homalea*, with the highest composition of BGCs (77), occurs from the immediate coast to many kilometers inland, forming soil, sand, and rock populations. Given these diverse ecological niches, *N. homalea* has already been reported to have a rich chemistry, including the depsides sekikaic or divaricatic acids detected by chemical analyses [57]. *Ramalina farinacea* is abundant on twigs and trunks, sometimes on rocks, and tolerant of air pollution and nutrient enrichment habitats [58] with 72 BGCs annotated. While *R. intermedia* is common on rocks in semi- or exposed habitats (71 BGCs, reference), *R. peruviana* is a pantropical species, mostly occurring in inland habitats (51 BGCs; [59]).

Ribosomally synthesized and posttranslationally modified peptides (RiPPs) are structurally complex naturally occurring metabolites but have been acknowledged only recently as a result of increasingly available genome sequencing data [60]. They are known from all three domains of life [61], but only minor hints of their occurrence in lichen fungi have been reported to the best of our knowledge [62]. Nevertheless, as RiPPs have rich and diverse bioactivities, and given that the annotated lichen RiPPs are phylogenetically rather different from the so far characterized fungal ones, the possible roles of RiPPs in lichens remain unclear. Further research is necessary to explore their functions.

## Figures and Tables

**Table 2:** The annotation for the T1PKS biosynthetic gene clusters from *T. dissimilis* with the putative function and similarity score to the MIBiG dataset.

Gene Tag/ antiSMASH Region	MIBiG Accession	% Similarity Score	MIBiG Compound Reference	UniProtKB/ Swiss-Prot Closest Reference	Putative Function (MIBiG)
***tondispred_002824*** ***(tondis_288)***	BGC0002161	71	1,3,6,8-tetrahydroxynaphthalene		Melanin biosynthesis
***tondispred_001459*** ***(tondis_191)***	BGC0000107	100	Naphthopyrone		Melanin biosynthesis
** R-PKS: ** ***tondispred_004967*** ** NR-PKS: ** ***tondispred_004968*** ***(tondis_787)***	BGC0000041	65	Oronofacic acid	A0A6F9DXA0.1	Menisporopsin A
***tondispred_004406*** ***(tondis_774)***	BGC0002175, BGC0000107	100	YWA1 (yellow pigment), Naphthopyrone		Melanin biosynthesis
***tondispred_004340 (tondis_683)***	BGC0001258	69	1,3,6,8-tetrahydroxynaphthalene	A7UMW1.1 Elsinochromes BGC, *PKS1*	Melanin biosynthesis

## Data Availability

The data supporting the findings of this study are available open access in figshare at http://doi.org/10.6084/m9.figshare.26417551. These include gff3 files and protein sequences from twenty-six re-annotated genomes downloaded from the NCBI. In addition, the alignment used for the KS tree calculation is deposited there. The de novo sequenced genome of *T. dissimilis* is available under the NCBI BioProject accession number PRJNA1142547 and on the Sequence Read Archive (SRA).
